# Screw placement through a higher medial portal provides better initial stability in arthroscopic ACL tibial avulsion fracture fixation: a finite element analysis

**DOI:** 10.1186/s12891-024-07695-5

**Published:** 2024-07-20

**Authors:** Yang Xiao, Changhao Shi, Geyang Deng, Zichu Ding, Jinhuang Xu, Bin Chen

**Affiliations:** 1 Division of Orthopaedics and Traumatology, Department of Orthopaedics, Nanfang Hospital, Southern Medical University, Guangzhou, China; 2 Department of Trauma and Joint Surgery, The Fourth Hospital affiliated to the Guangzhou Medical University, Guangzhou, China

**Keywords:** ACL avulsion fracture, Arthroscopic screw fixation, Portal, Biomechanical effect, Finite element analysis

## Abstract

**Objective:**

The objective of this study was to investigate the initial stability of different screw placements in arthroscopic anterior cruciate ligament (ACL) tibial avulsion fracture fixation.

**Methods:**

A three-dimensional knee model at 90° flexion was utilized to simulate type III ACL tibial avulsion fracture and arthroscopic screw fixation through different portals, namely the central transpatellar tendon portal (CTP), anterolateral portal (ALP), anteromedial portal (AMP), lateral parapatellar portal (LPP), medial parapatellar portal (MPP), lateral suprapatellar portal (LSP), medial suprapatellar portal (MSP). A shear force of 450 N was applied to the finite element models at 30° flexion to simulate the failure condition. The displacement of the bony fragment and the volume of the bone above 25,000 µ-strain (damaged bone volume) were calculated around the screw path.

**Results:**

When the screw was implanted through CTP, the displacement of the bony fragment reached the maximum displacement which was 1.10 mm and the maximum damaged bone volume around the screw path was 148.70 mm^3^. On the other hand, the minimum displacement of the bony fragment was 0.45 mm when the screw was implanted through LSP and MSP. The minimum damaged bone volume was 14.54 mm^3^ around the screw path when the screw was implanted through MSP.

**Conclusion:**

Screws implanted through a higher medial portal generated less displacement of the bony fragment and a minimum detrimental strain around the screw path. The findings are clinically relevant as they provide biomechanical evidence on optimizing screw placement in arthroscopic ACL tibial avulsion fracture fixation.

## Introduction

Anterior cruciate ligament (ACL) tibial avulsion fractures are relatively rare injuries that occur mostly in children and adolescents aged 8–14 [1]. These injuries can also occur in adults and are equivalent to acute ACL ruptures [2]. Avulsion fractures commonly involve the intercondylar depression, where the ACL insertion lies. The fracture has been classified into three types (type I, II and III) according to the severity of displacement by Meyers and McKeever [3]. Zaricznyj applied this classification with a type IV for comminuted fractures [4]. Conservative treatment results are ineffective for type III fractures, so surgical operation should be considered [5, 6]. Arthroscopic reduction and fixation with screws or sutures are the mainstream treatment for these fractures unless other lesions require open surgery [2].

Assessment of the clinical outcome of screw and suture fixation remains ambiguous. According to the latest systematic review and meta-analysis, there are no significant differences in clinical outcome scores between the two approaches [7]. Screw fixation is relatively simple and allows the surgeon to maintain compression of the fracture fragments, whereas the suture fixation has a decreased risk of implant removal [8]. A single 3.5–4.0 mm screw is sufficient to maintain the bony fragment in place in the case of arthroscopic screw fixation [9–11]. Screws are usually implanted at a high knee flexion angle [10, 11]. The objective of ACL avulsion fracture treatment is to restore the isometry and tension of the ACL by anatomic reduction and fixation. Malunion and prolonged knee instability may be caused by the potential displacement of the bony fragment, even requiring revision surgery [9, 12–14]. Therefore, studying the initial stability of the fixed ACL complex after surgery is necessary. The screw has been reported in previous clinical or biomechanical studies to be implanted through a particular portal or the dissected knee joint [9–11, 15–17]. The influence of screw placement through different portals on the biomechanical behaviour of the fixed ACL complex is little understood. However, different portals will change the orientation of the screw in the bone, which may affect the stability of the bony fragment under the tension of the ACL. Understanding the biomechanical differences caused by the surgical portal could provide the foundation for optimizing surgery. This study aims to investigate the initial stability of different screw placements in arthroscopic ACL tibial avulsion fracture fixation.

Finite element models were developed to simulate the failure condition and evaluate the displacement of the bony fragment and the strain around the screw path in this study. The strain above 25,000 µ-strain was considered as fracture strength of the bone [18]. To the knowledge of the authors, this is the first study to explore the biomechanical stability of screw placement orientation in ACL tibial avulsion fracture fixation. The hypothesis was that screw insertion through a higher medial portal would offer better initial stability, resulting in less displacement of the bony fragment and a minimum detrimental strain surrounding the screw path.

## Materials and methods

### Establishment of the three-dimensional (3D) models

The 3D models from the previous work of the authors were employed in the present study as well [19]. The right knee of a healthy volunteer was imaged using computed tomography (CT) (SOMATOM Definition AS+; Siemens) with a thickness of 0.6 mm and resolution of 512 × 512 pixels. The 3D models of the femur and tibia were reconstructed from the CT images in Mimics (v19.0, Materialize NV, Leuven, BE). The knee models at 30° and 90° flexion were obtained by matching their outlines to the corresponding fluoroscopic images during a lunging motion.

### Surgical modelling

**Fig. 1:**
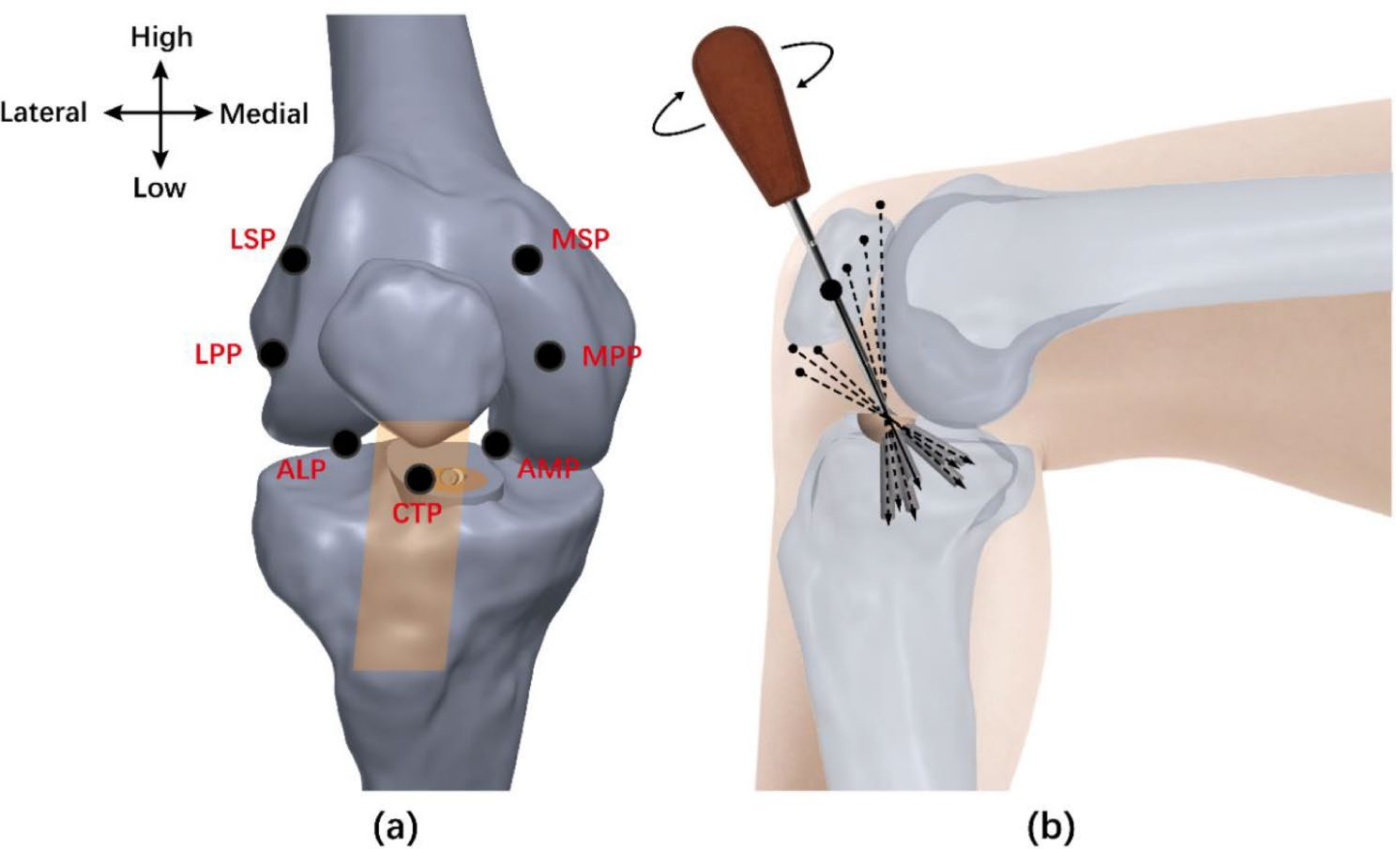
(**a**) Identification of the arthroscopic portals of the knee joint (*CTP* central transpatellar tendon portal; *ALP* anterolateral portal; *AMP* anteromedial portal; *LPP* lateral parapatellar portal; *MPP* medial parapatellar portal; *LSP* lateral suprapatellar portal; *MSP* medial suprapatellar portal; *orange parallelogram* patellar tendon). (**b**) The various screws were implanted through different portals

Seven distinct arthroscopic portals around the patella were identified on the knee model at 90° flexion (Fig. 1a). The central transpatellar tendon portal (CTP) was discovered in the centre of the patellar tendon just under the inferior edge of the patella. The anterolateral portal (ALP) and anteromedial portal (AMP) were aligned with the inferior edge of the patella on both sides of the patellar tendon. The lateral parapatellar portal (LPP) and medial parapatellar portal (MPP) were located 5 mm from the lateral and medial edge of the patella. The lateral suprapatellar portal (LSP) and medial suprapatellar portal (MSP) were identified at the junction of the tangential line between the superior and medial/lateral edges of the patella.

The tibial and femoral footprint of the ACL were determined by the bony landmarks as described in the previous article [19]. The bony fragment was located on the position of ACL tibial insertion. A hemispherical bony fragment with a diameter of 12 mm was designed to ensure the orientation of the screw as the sole variable. All insertion points of the screws were the same, located in the centre of the bony fragment. According to a previous study, a screw should not be larger than one-third of the diameter of the bone fragment to prevent comminution [20]. Therefore, each screw was designed with a diameter of 3.5 mm and a length of 28 mm. The orientation of the screw was dictated by the position of the portal and the insertion point of the screw. Figure 1b shows the various screws through different portals. All modelling procedures were performed using SolidWorks (v2018, Dassault Systemes, Massachusetts, USA).

### Finite element models of the knee joint

The 3D models were exported into the finite element analysis software Abaqus (v2018; Dassault Systèmes SE, Vélizy-Villacoublay, FR). The femur was assumed a shell and defined as a rigid body. The bony fragment and tibia were assumed solids and were defined as a homogeneous isotropic elastic material with Young’s modulus E = 389 MPa and Poisson’s ratio v = 0.3 [21]. The material of the screw was stainless steel and was defined as a homogeneous isotropic material with Young’s modulus E = 205 GPa and Poisson’s ratio v = 0.3 [22]. The ACL was assumed to be solid and was defined as a homogeneous isotropic elastic material with Young’s modulus E = 345 MPa and Poisson’s ratio v = 0.45 [23]. A free meshing technique was applied for the entire model (femur: four-node 3-D bilinear rigid quadrilateral element, element type: R3D4; bony fragment and tibia: four-node linear tetrahedron element, element type: C3D4; screw and ACL: eight-node linear hexahedral element, element type: C3D8).

**Fig. 2:**
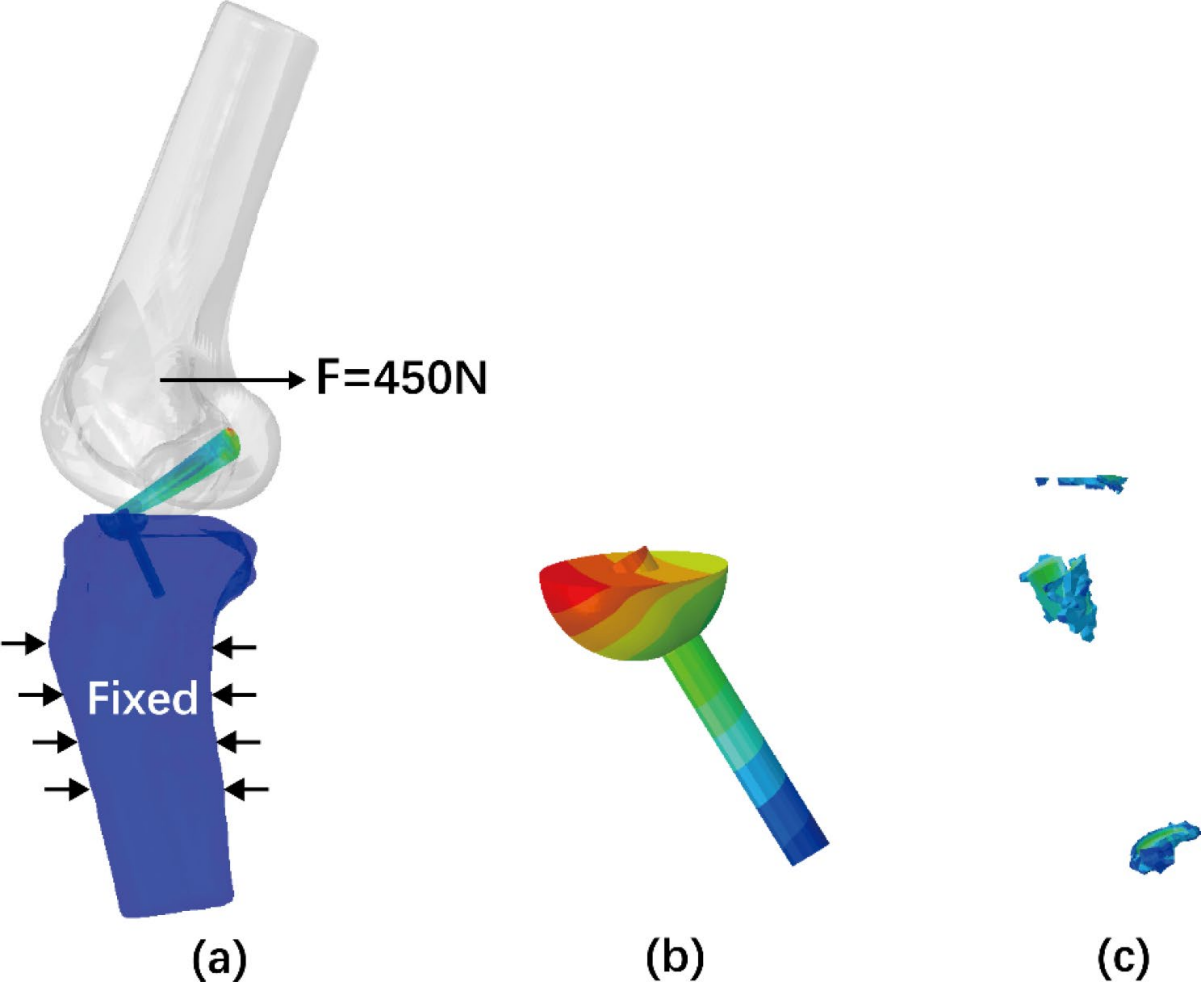
(**a**) The mid-lower part of the tibia was fixed and a posterior shear force was applied at the femur at 30° of knee flexion; (**b**) The maximum displacement of the fixed bony fragment was calculated. (**c**) The volume of strain on the bone above 25,000 µ-strain was recorded

Bonded contact between the ACL and the femur and between the ACL and the top face of the bony fragment were defined. For both models, the screw-bone contact interfaces and the bony fragment-tibia contact interfaces were modelled as sliding interactions with a friction coefficient of 0.3 [24]. The screw tightening preload of 300 N was used to tighten the screw. The compression effect of the screw on the bony fragment was accurately simulated by defining a bonded contact between the screw head and the top face of the bony fragment. The mid-lower part of the tibia was fixed. Studies have reported that the fixed ACL complex was loaded in the range of 30–450 N for the rehabilitation process, depending on the activity [25–27]. A posterior shear force of 450 N was applied to the femur at 30° of knee flexion to simulate an anterior tibial displacement (Fig. 2a). The displacement of the bony fragment and the strain around the screw path were subsequently calculated. The volume of strain above 25,000 µ-strain on the bone was recorded (Fig. 2b, c).

### Validation of finite element models

A finite element model of arthroscopic screw fixation through AMP was first established and analysed. To perform mesh sensitivity analysis, stepwise upsizing on the mesh size was applied [28]. The convergence tolerance was set as a variation of stress within 5% from the previous model with higher mesh density. The finally used mesh size of the screw, the bony fragment and the ACL were 0.5 mm, 0.5 mm and 1.0 mm, respectively. The mesh size of the tibia was 1.5 mm, and mesh refinement was performed on the 0.5 mm contact with the bony fragment and screw (Table 1). The initial displacement of the bony fragment was compared to the literature results [15–17] (Table 2).

**Table 1:** Numbers of nodes and elements of the four components

Components	Nodes	Elements
Screw	4,389	3,640
Bony fragment	7,431	37,172
ACL	3,367	2,772
Tibia	70,118	379,772

**Table 2:** Comparisons of initial displacement of the bony fragment in the present study and previous studies

Study	Applied maximum load to failure (*N*)	Initial displacement (mm)
Present study	450	0.88
Ezechieli et al. [16]	311.7 ± 120.3	0.84 ± 0.15
Eggers et al. [15]	457.1 ± 13.4	2.17 ± 1.4
In et al. [17]	101.8 ± 29.0	0.4 ± 0.2

**Fig. 3:**
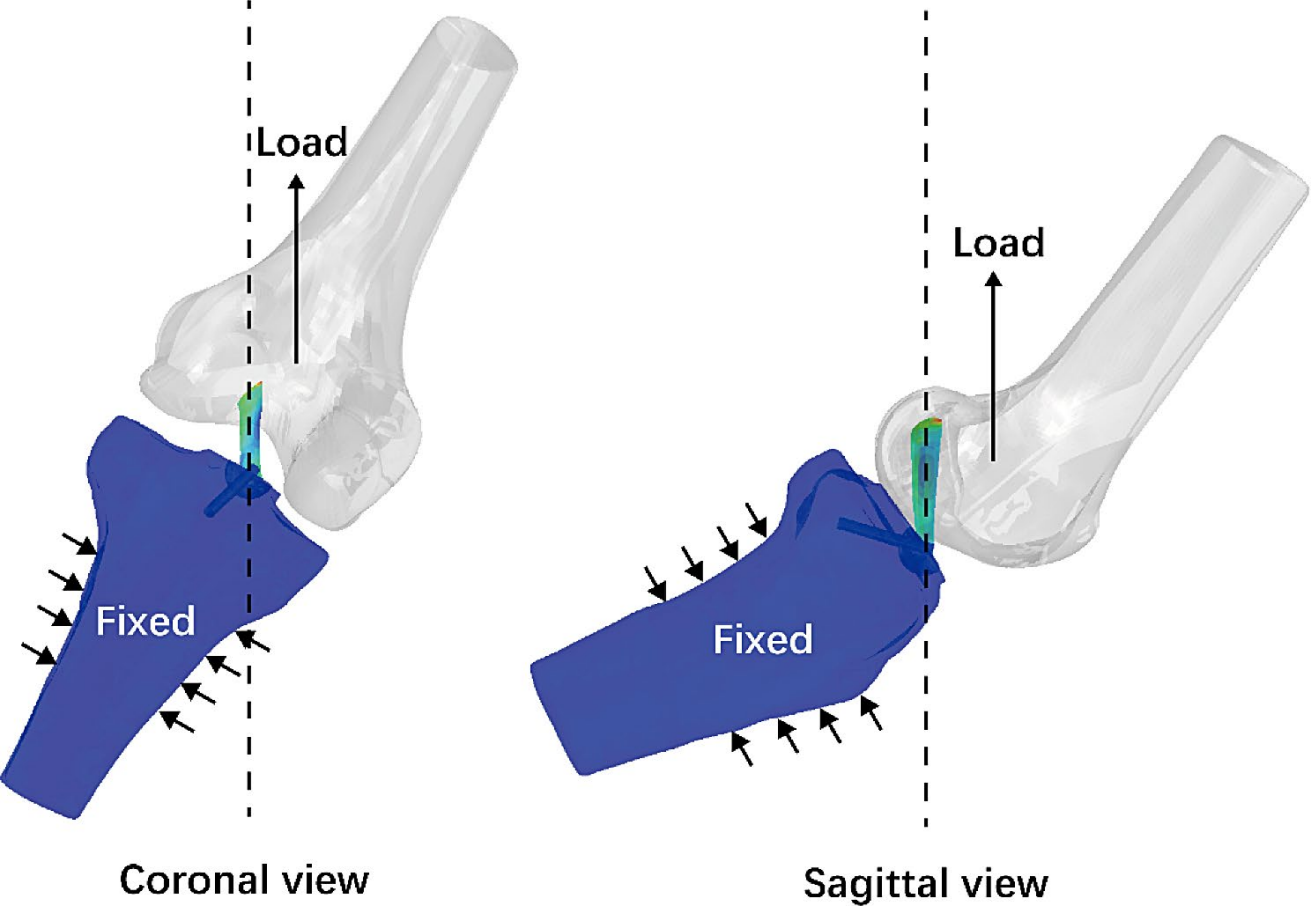
Schematic of tensile testing. The tensile load was applied along the axis of the ACL while the normal anatomical angles of ACL were preserved

**Fig. 4:**
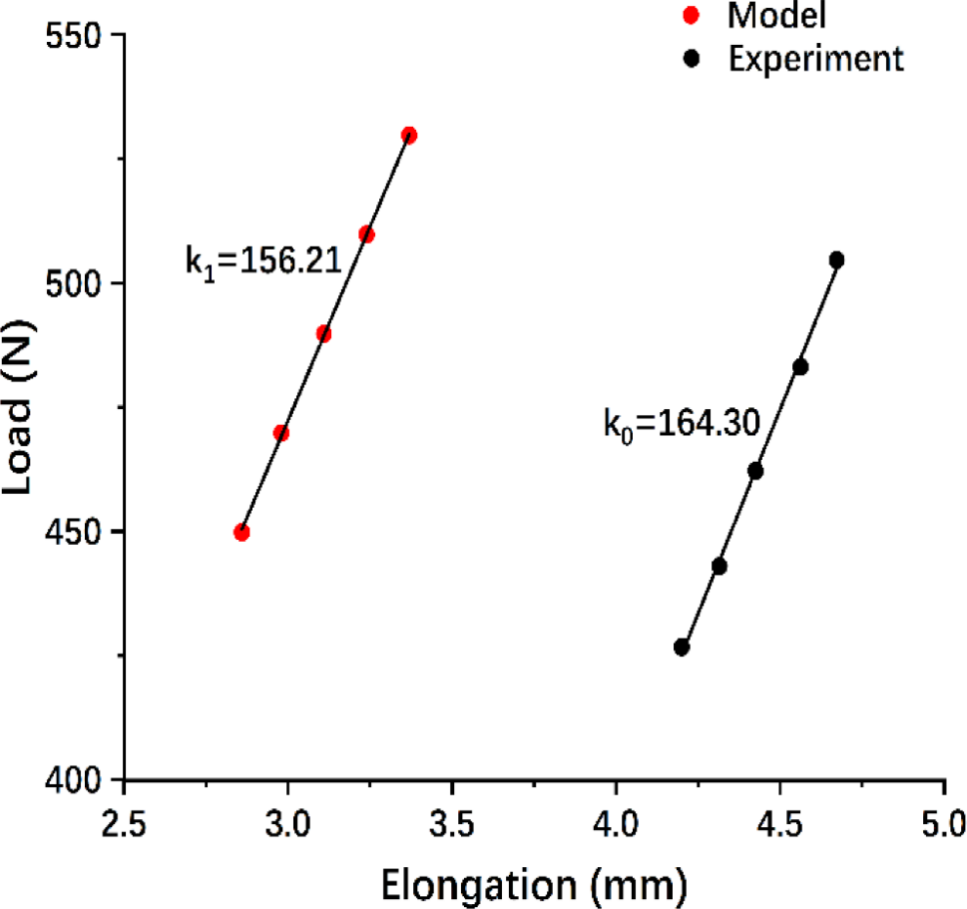
Load-elongation curves for tensile testing demonstrate the similarities in results between the model and experiment

To validate the results obtained through finite element analysis, a tensile testing simulation was performed according to the previous mechanical study [29]. The tibia-ACL-femur model was applied to a tensile load along the axis of the ACL at 30° of knee flexion (Fig. 3). The tensile load was beginning at 450 N and incrementally increased by 20 N up to 530 N. The load-elongation curve was constructed, and the angular coefficient of the linear equation was compared to the experimental result (Fig. 4). The results of comparison showed that a variance of 4.92% exists between the finite element analysis and mechanical test within the discrepancy threshold of 20% [30]. Therefore, the finite element model can be used in subsequent research, as it exhibits similar structural properties to mechanical testing.

## Results

**Fig. 5:**
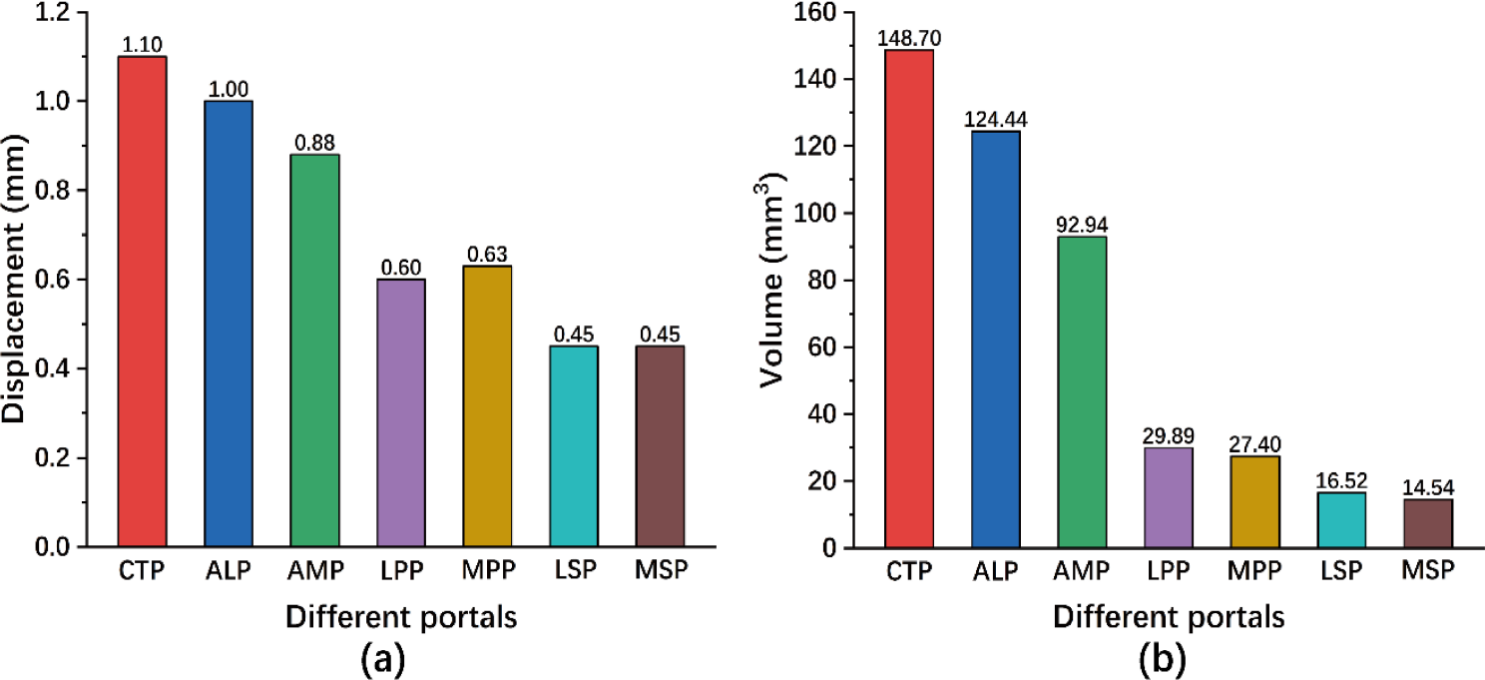
Comparison of **(a)** the maximum displacement of the bony fragment and **(b)** the volume of strain on the bone above 25,000 µ-strain among the different portals

In both medial (MLP, MPP and MSP) and lateral portals (ALP, LPP and LSP), the displacement of the bony fragment gradually decreased when portals were located higher. The maximum displacement of the bony fragment was 1.10 mm when the screw was implanted through CTP. On the other hand, the minimum displacement of the bony fragment was 0.45 mm when the screw was implanted through LSP and MSP. The average displacement of the bony fragment was 0.68 mm and 0.65 mm, for the lateral and medial portals, respectively (Fig. 5a).

**Fig. 6:**
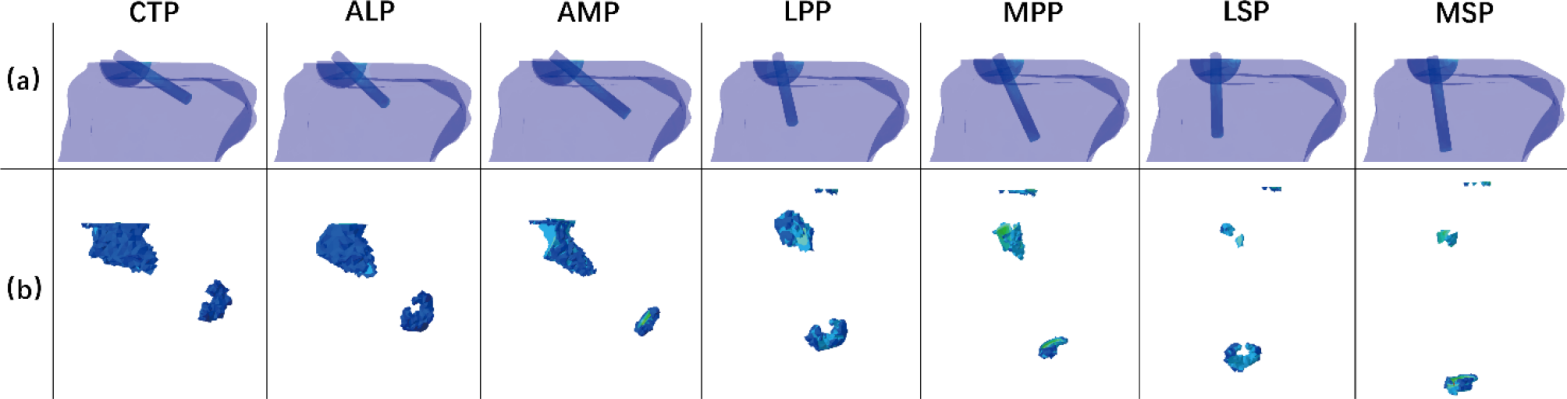
The distribution of the damaged bone for each model. (**a**) The position of the screw in the bone from the sagittal view; (**b**) The damaged bone around the screw path

In both medial and lateral portals, the damaged bone volume decreased when portals were located higher. The maximum volume of strain above 25,000 µ-strain on the bone was 148.70 mm^3^ when the screw was implanted through CTP. Correspondingly, the minimum volume of strain above 25,000 µ-strain on the bone was 14.54 mm^3^ when the screw was implanted through MSP. For the lateral and medial portals, the average volume of the damaged bone was 56.95 mm3 and 44.96 mm3, respectively (Fig. 5b). The distribution of the damaged bone for each model is shown in Fig. 6.

**Fig. 7:**
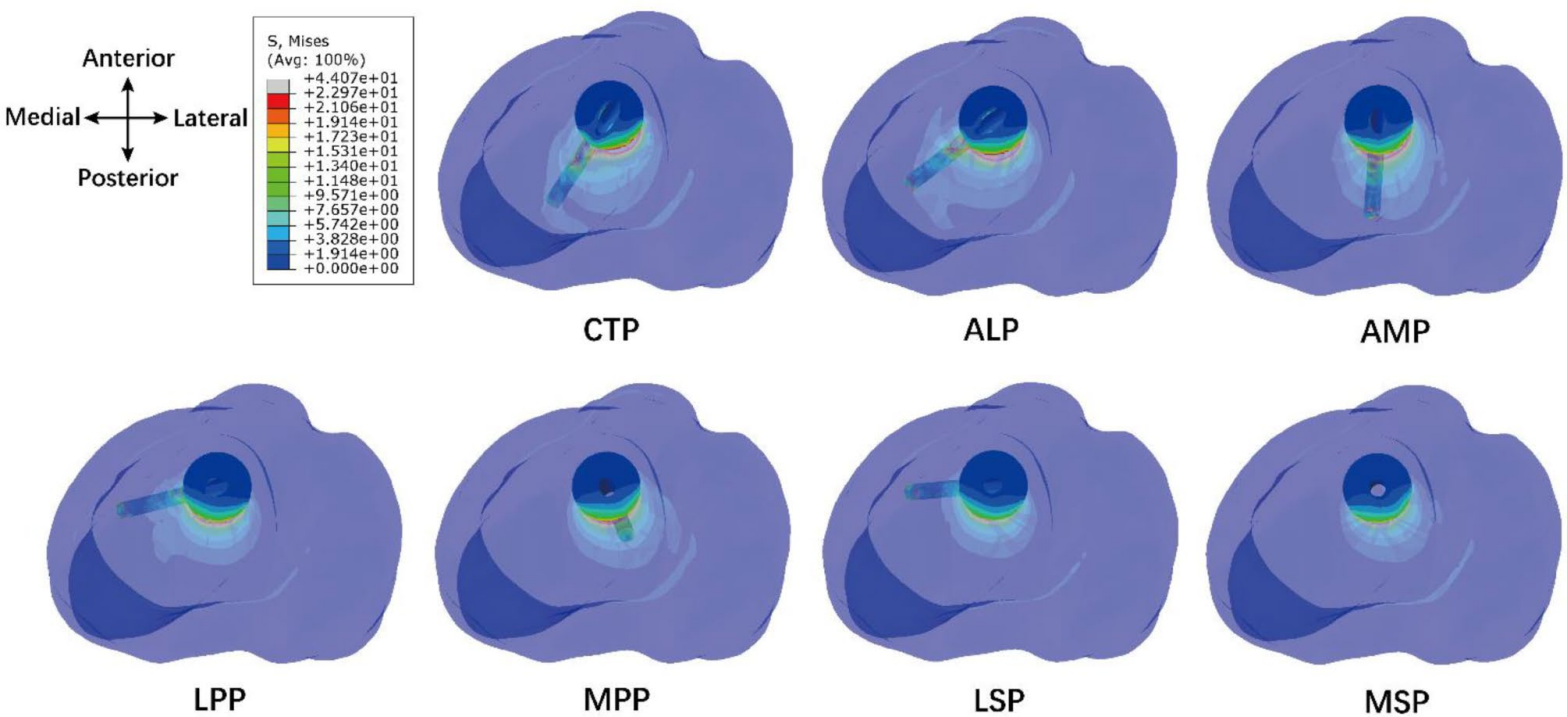
Distribution of Mises stress on bony fragment and tibia through a view perpendicular to the tibial plateau

Figure 7 showed the stress distribution on the bone for each model. Various stress distribution patterns existed due to the different screw insertions. The stress concentration point on the bony fragment occurred at the posterior contact surface between the two bones in all models. The stress concentration point on the tibia occurred at the end of the screw in LPP and LSP models, occurred at the posterior contact surface between the two bones in MPP and MSP models, and occurred at the junction between the two bones and screw in CTP, ALP and AMP models. The maximum Mises stresses were 33.00 MPa and 44.07 MPa on the bony fragment and the tibia, respectively, when the screw was implanted through CTP. The minimum Mises stresses were 16.25 MPa and 22.97 MPa on the bony fragment and the tibia, respectively, when the screw was implanted through LSP and MSP.

## Discussion

The most noteworthy finding of the present study was that screws implanted from different portals could impact the displacement of the bony fragment and the strain on the bone around the screw path. Screws that were implanted through a higher medial portal resulted in less displacement of the bony fragment and a minimum damaging strain around the screw path. The current findings are of clinical relevance as they might provide biomechanical evidence to optimize screw placement in arthroscopic ACL tibial avulsion fracture fixation.

The initial stability is essential to the risk of residual laxity and could predict potential loss in reduction during healing after surgery [31]. Previous research on biomechanics has focused on comparing the strength of screw fixation and suture techniques [15, 31–34]. Sometimes studies favour either screws or sutures, while other times reporting no significant difference between them. Eggers et al. [15] reported that a second screw has no positive effect on the biomechanical characteristics than one-screw fixation. Regarding the method of screw placement, Ezechieli et al. [16] and In et al. [17] used the dissected knee joint to implant the screw, which was inconsistent with the condition of arthroscopic screw fixation. As for the arthroscopic portal for screw implanting, Hunter et al. [9] implanted the screw through CTP and Lubowitz et al. [10] through AMP. Senekovic et al. [11] implanted the screw through a superior anteromedial portal, higher than AMP and lower than MPP. However, there is no consensus on the effect of different portals on the biomechanical stability of the screw. To the knowledge of the authors, this is the only study to explore the biomechanical stability of screw placement orientation in ACL tibial avulsion fractures. The results showed that CTP might not be a good choice when implanting the screw with the arthroscopic technique. Instead, it might be better to implant the screw through a higher medial portal as much as possible.

Experiments in previous biomechanical studies were performed at specific knee flexion angles, approximately in the Lachmann position [15, 34]. Cyclical loading forces such as anterior shear or upward traction force were applied to the tibia or femur to simulate the failure conditions [15, 17]. In the current study, shear loads were applied at 30° of knee flexion to be consistent with in vitro experiments. Standardized cubic bony fragments with various sizes have usually been created in previous studies to simulate the ACL tibial avulsion fracture [17, 32]. Considering the experimental rigour, hemispherical bony fragments were hereby used to ensure that the screw orientation was the only variable, although it was inconsistent with the clinical fracture pattern. In addition, simplification of tissue properties was performed in this study, which would affect the magnitude but not the tendency of calculated results. Even so, validation results showed that the structural properties and elastic deformations of the model were reliable.

The pull-out of the screw and fracture of the fragment were two main failure modes in previous literature, with the former one being the most common. Ezechieli et al. [16] reported 60% screw pull-out failure and 40% fragment fracture failure. In et al. [17] reported 86% screw pull-out failure and 14% fragment fracture failure. Bong et al. [32] found the single observed failure mode for the specimens was screw pull-out from the cancellous bone of the fracture bed. Unlike other failure modes of fracture internal fixation, none of the literature studies has reported screw breakage in treating ACL tibial avulsion fractures. Feng et al. [35] indicated that screw loosening is closely related to bone damage caused by abnormal stress around the screw. That was the reason why this study investigated the damaged bone volume around the screw path. The damaged bone was mainly concentrated at the junction of the bony fragment and the tibia and the end of the screw, which could reduce the stability of the screw (Fig. 6). The smaller the damaged bone volume, the less probability the screw would pull out from the bone.

This study has several limitations. First, only one direction of force was applied, and the cyclical loading force from daily knee motion, such as walking, was not analyzed. In fact, the force of the ACL is quite complex, depending on different activities. Additionally, the bony fragment is idealized as a hemisphere, which is inconsistent with the actual situation. The simplification to be able to control the single variable is necessary. The thread of the screw was not considered, and only one size of the screw was explored in this study, which is not entirely consistent with clinical practice. However, different screw sizes may affect the biomechanical effect of screw fixation. Since this study was mainly designed to explore the impact of different surgical portals on the initial stability of screw fixation, these factors may not affect the conclusions. Furthermore, the current study did not take into account the growth plate presence, which might challenge the surgeon’s decision to select the portal when treating children and adolescents.

## Conclusion

The findings of this study demonstrate that screw insertion through a higher medial portal improves the initial stability in arthroscopic ACL tibial avulsion fracture fixation, resulting in less displacement of the bony fragment and a minimum detrimental strain surrounding the screw path. Although there is no clear advantage of screw fixation compared with suture fixation in the treatment of ACL tibial avulsion fracture. The results are clinically relevant as they provide biomechanical evidence on optimizing screw placement in arthroscopic ACL tibial avulsion fracture fixation.

## Data Availability

The data that support the findings of this study are available from the corresponding author upon reasonable request.

## Abbreviations

ACL: Anterior Cruciate Ligament
CTP: Central Transpatellar tendon Portal
ALP: Anterolateral Portal
AMP: Anteromedial Portal
LPP: Lateral Parapatellar Portal
MPP: Medial Parapatellar Portal
LSP: Lateral Suprapatellar Portal
MSP: Medial Suprapatellar Portal
3D: Three-Dimensional
CT: Computed Tomography
